# Rumen-protected methionine a feed supplement to low dietary protein: effects on microbial population, gases production and fermentation characteristics

**DOI:** 10.1186/s13568-019-0815-4

**Published:** 2019-06-26

**Authors:** Imtiaz Hussain Raja Abbasi, Farzana Abbasi, Lihui Liu, Bello M. Bodinga, Mervat A. Abdel-Latif, Ayman A. Swelum, Mohamed Abdalla Elsiddig Mohamed, Yangchun Cao

**Affiliations:** 1 College of Animal Science and Technology, Northwest A&F University, Yangling, Shaanxi China; 2 Department of Animal Nutrition, Faculty of Animal Production and Technology, Cholistan University of Veterinary and Animal Sciences, Bahawalpur, Punjab Pakistan; 3 School of Life Science and Engineering, Southwest University of Science and Technology, Mianyang, Sichuan China; 4 Department of Nutrition and Veterinary Clinical Nutrition, Faculty of Veterinary Medicine, Damanhour University, Damanhour, 22511 Egypt; 5 Department of Theriogenology, Faculty of Veterinary Medicine, Zagazig University, Zagazig, 44519 Egypt

**Keywords:** Dietary protein, Feed degradability, Fermentation, Rumen-protected methionine, Rumen simulation

## Abstract

The present study was performed to evaluate the effects of different concentration of rumen-protected methionine (RPMet) with a low level of crude protein (CP) using rumen simulation technology on many parameters. The experiment was assigned randomly into four treatments: (1) high protein diet (163.39 g/kg CP) without RPMet (HP); (2) low protein diet (146.33 g/kg CP) without RPMet (LP); (3) low protein diet, supplement with low RPMet (RPMet: 0.11 g/kg) (LPLMet); and (4) low protein diet, supplement with high RPMet (RPMet: 0.81 g/kg) (LPHMet), mixed with 20 g basal diet in each fermenter. Based on National Research Council (NRC) (Nutrient requirements of dairy cattle, National Academies Press, Washington, DC, 2001) recommendation for dairy ruminants HP diet was formulated as positive normal control and LP as a negative control. Results demonstrated that CP disappearance was found significantly higher (P < 0.05) in supplement groups compared with HP and found similar (P > 0.05) with LP. However, neutral detergent fiber (NDF) and gross energy (GE) were found a parallel among supplement groups compared to HP and higher than LP. Furthermore, microbial crude protein, total and short chain fatty acids were found similar in LPHMet and HP and found significantly higher than LPLMet and LP. The *R. albus* population of LPHMet found parallel to HP and pointedly higher than LP in a solid and liquid fraction. Daily production of ammonia nitrogen, total gas, and methane were higher in HP than LP, LPLMet, and LPHMet. Overall, results concluded that values of digestibility, rumen fermentation, microbial crude protein, and *R. albus* population were similar of LPHMet to that of HP group. However, production of ammonia-N, total gas, and methane volume were significantly higher in the HP group than LPLMet, LPHMet, and LP groups. In conclusion, rumen-protected methionine is a good feed supplement to low dietary protein in the level of 0.81 g/kg.

## Introduction

Rumen environment quickly degrades free amino acids (AA) and could not maintain the performance of dairy ruminants. The ruminants obtain AA mainly from two sources microbial protein and rumen undegradable protein (RUP) that is washed to the abomasum and appear to meet the maintenance needs of ruminants, these both play a significant role to keep the balance of metabolizable protein (MP) of ruminants. These sources must be absorbed as free AA from the small intestine of ruminants (Faciola and Broderick 2014). Rumen-protected methionine (RPMet) known as top limiting essential AA for ruminants, particularly in those where milk is sold not only for volume but also for the component values (Vasconcelos et al. 2007; Abbasi et al. 2018a). Its supplementation could improve the AA balance of MCP and consequentially reduce the deamination of absorbed AA and decrease blood urea nitrogen which is valuable for the reproduction and performance of dairy animals (Rhoads et al. 2006). Increasing the level of limiting amino acids in the small intestine, resulting in increasing milk volume, protein, fat yields and reduces urine nitrogen (Zhang et al. 2013; Abbasi et al. 2017). Amino acids balance is considered as the most significant factor compared to the supplementation of total rumen degradable protein (RDP) for improving the nitrogen utilization. Limiting AA flow particularly methionine in the small intestine is considered the essential element for milk yield and milk protein production (Noftsger et al. 2005). It became a growing objective to reduce crude protein (CP) in animal diets, with the supplementation of rumen-protected limiting amino acids to raise the quantity of MP as it increases AA flow in the small intestine. And decrease nitrogen losses, feed cost and global environmental pollution, without adverse impact on animal performance (Sinclair et al. 2014; Guyader et al. 2016).

Therefore, it is imperative to evaluate and compare the effects of supplementation of RPMet systematically with low dietary protein diet in modulating ruminal fermentation. To this end, the current study was to determine the degree to which level RPMet supplementation could replace low protein diet concerning their effects on rumen fermentation, nutrients metabolism, gases production, microbial protein synthesis and specific bacterial population using rumen simulation technique (RUSITEC). Because, high dietary CP not only increase the heat increment with energy loss but also led to environmental problems; with “low CP diet + RPMet” feed cost, energy losses, as well as nitrogen losses can be decreased.

## Materials and methods

### Experimental apparatus and description of the test product

The experiment was carried out using a rumen simulation technique (RUSITEC, Sanshin, Tokyo, Japan) as described by Kajikawa et al. (2003). The test product was provided as mepron^®^ is a rumen-protected dl-methionine 85.00% (CAS-RN: 59-51-8; dl-methionine) and rumen bypass rate was 80.00% developed by Evonik (Germany Industries AG, health, and nutrition feed additives Rodenbacher Chaussee 4-63457 Hanau-Wolfgang).

### Donor animals and experimental diets

Three healthy Xinong Saanen goats [average initial body weight (50.63 ± 1.20 kg)], were housed in pens and the same basal RUSITEC diet offered as a total mixed ration to animals at 07:00 and 19:00 with free access to water. Rumen contents were collected through the ruminal fistula before the morning feeding and placed into thermos flasks pre-heated at 39 °C by filling with hot water and quickly transferred to the laboratory. The experiment was randomly designed and conducted over two independents 15 days incubation periods with 7 days for adaption and 8 days for samples collection. The four groups were: (1) high protein without RPMet (HP, 163.39 g/kg CP), (2) low protein without RPMet (LP, 146.33 g/kg CP), (3) low protein (141.80 g/kg CP) supplemented with low concentration of RPMet (0.11 g/kg DM) (LPLMet), and (4) low protein (143.30 g/kg CP) supplemented with a high concentration of RPMet (0.81 g/kg DM) (LPHMet). Based on NRC (2001) recommendation of CP for dairy cows, HP diet was formulated as a positive control, LP as a negative control group. The chemical compositions of experimental diets were similar among all treatments without out CP percentage, and other formulated values are represented in Table 1.

**Table 1:** Ingredients and chemical composition of diets offered to animals and used in RUSITEC experiment (dry matter basis)

Items	Treatments				SEM	P-value
	HP	LP	LPLMet	LPHMet		
Ingredients, g/kg dry matter						
Corn silage	182.40	193.60	193.60	193.50		
Alfalfa hay	101.60	39.20	39.20	39.20		
Wheat straw	153.00	213.80	213.70	213.60		
Ground corn	162.30	216.50	216.50	216.40		
Wheat bran	77.80	46.80	46.80	46.70		
Soybean meal	70.30	100.50	100.50	100.40		
Cottonseed meal	96.20	38.20	38.20	38.20		
Corn germ meal	115.50	105.60	105.60	105.60		
Limestone	10.50	8.80	8.80	8.80		
Di calcium phosphate	0.00	4.80	4.80	4.80		
Sodium chloride	10.50	12.00	12.00	12.00		
Sodium bicarbonate	1.90	2.00	2.00	2.00		
Premix*	18.00	18.10	18.10	18.00		
RPMet	0.00	0.00	0.11	0.81		
Chemical composition						
Dry matter (g/kg DM)	902.50	890.25	892.00	893.75	5.40	0.89
Crude protein (g/kg DM)	163.39^a^	146.33^b^	141.80^b^	143.30^b^	3.38	0.01
Neutral detergent fiber (g/kg DM)	471.16	471.72	471.21	470.38	1.28	0.99
Acid detergent fiber (g/kg DM)	212.12	210.87	205.37	212.27	2.04	0.70
Ether extract (g/kg DM)	28.50	28.00	28.02	28.11	0.52	1.00
Gross energy (MJ/kg DM)	16.28	16.13	16.13	16.50	0.14	0.85

SEM, standard error of the mean

^a,b^Superscripts values within the same row, are significantly different at (P < 0.05)

* Premix (per kilogram of total-mixed ration, DM basis contains): 10.5 mg Cu, 9.80 mg Zn, 12.00 mg Mn, 0.11 mg. Co, 0.32 mg I, 0.15 mg Se, 2500 IU vitamin A, 500 IU vitamin D3, and 40 IU vitamin E

### Experimental procedure and sampling

After collection of rumen inoculum, the inoculum was mixed, strained through four layers of cheesecloth maintained anaerobic condition. Incubation was started as described Zhao et al. (2010). On day 1st each fermenter was filled with 350 mL strained liquid inoculum with 350 mL of artificial saliva solution as described by McDougall (1948). One nylon bag containing 70 ± 0.05 g of solid rumen digesta (wet weight basis) as inoculum and one nylon bag of an experimental diet comprising roughages and concentrates (45:55 DM, basis) weighted 20 ± 0.04 g. To increase the chewing activity of ruminants the roughages and concentrates ingredients was grounded to pass through (4 and 2 mm) sieves respectively. To maintained and improve the flow of microbes in fermenters, the size of nylon bags was selected 18 × 9 cm with a pore size of 100 μm. To maximize the ruminal peristalsis and increase ruminant’s salivation secretion, each fermenter in vertical position were agitated at 2 cycles/min and artificial saliva (McDougal’s) was freshly prepared and infused continuously into each fermenter by precision pump guaranteed at a rate of approximately 2.85%/h directed by Kajikawa et al. (2003). After 24 h solid inoculum bag was replaced with a new feed bag. On subsequent days, the old bag 48 h replaced with new one. Consequently, each bag incubated in its fermenter for 48 h and the CO_2_ flush was maintained during the entire process. After the adaption period, on days 8, 9, and 10, the 5 mL fluid was subsampled from each fermenter to determined pH immediately before exchanging the feed bags. Three litter Tedlar bags were put into the inlet of each fermenter and closed with a plastic screw to collect gas during 24 h from RUSITEC fermentation to determine the concentration of methane, hydrogen, and carbon dioxide gases production. After the adoption period, the 20 mL of H_2_SO_4_ (3.66 M, 20%, V/V) was added to each effluent bottle to control further fermentation. The 6 mL effluent liquid was collected for analysis of volatile fatty acid (VFA) mixed with 1 mL of 25% of metaphosphoric acid, and 5 mL of effluent was preserved to analyze ammonia-N (NH_3_-N) concentration and stored under − 20 °C for analysis. One feed bag (containing residue) from every fermenter was collected, clean with 100 mL of artificial saliva then washed into the cold rinse cycle for (10 min) using the washing machine, dried in air forced oven for 24 h at 65 °C stored at room temperature for further nutrients disappearance analysis. On 11 days, after replacing the feed bag from each fermenter 6 mL fluid was collected and pH was measured immediately at 0, 2, 4, 6, 8, 12, 18 and 24 h. On 12 and 13 days, 5 mL of saturated HgCl_2_ was added to the effluent collection bottles, all bottles were held in an ice bath. On 13 and 14 days, approximately 400 mL of effluent fluid was collected for isolation of liquid associated microbial mass, and nylon bags contents were preserved for solid associated microbial mass to examine microbial crude protein (MCP) concentration. On day 15, during 24 h incubation period by the displacement of water, total gas was collected in 3 L Tedlar bags and gas volume was measured. Additionally, 10 mL of fermenters fluid as the liquid fraction and one feed bag contents covering undigested feed after 48 h incubation as the solid portion from each fermenter was freeze-dried for DNA study of total bacteria and specific cellulolytic species analysis.

### Laboratory analyses of feed and fermenters samples

Feed and RUSITEC residue samples were dried at 105 °C for 24 h and ground to pass through a 1 mm screen (Standard model 04, Arthur Thomas, Philadelphia) for further chemical analysis. The CP content was analyzed using micro Kjeldahl (Foss, Dk, 3400 Hillerod, Denmark). Acid detergent fiber (ADF) and neutral detergent fiber (NDF) values were determined as described by VanSoest et al. (1991) with the modified method used with an ankom^200^ fiber analyzer unit (Ankom Technology, Macedon, USA). Alpha-amylase (Sigma A-3306, Sigma, Aldrich, China), and sodium sulfite were added to each sample separately for NDF determination. Both ADF and NDF were expressed inclusive of residual ash, and all samples were analyzed in triplicate. Ether extract (EE) was examined by extracting samples with petroleum ether using a Gerhardt Soxtherm-2000 Automatic (AOAC, 1990; id 920.39). Gross energy (GE) of samples was determined by complete oxidation using an adiabatic bomb calorimeter (AC 500, Leco, St. Joseph, and Mi) (Parr Instrument Co, 1970). The dietary RPMet ratio of each ration was estimated based on the included ingredients using the Cornell, Penn-Miner Dairy (CPM, Dairy, and Version 3.0.8.1) Software. The total and individual VFA concentrations of each sample were determined through gas chromatography (Agilent Technology 7820A GC system, Santa Clara, USA) using a 30 m × 0.25 mm × 0.33-m fused silica column (Atech Tech, Co., Ltd. PR, China) followed the procedure of Abbasi et al. (2018b). The concentration of gases was analyzed by gas chromatography (model 663-30, Hitachi Corporation, Tokyo, Japan) equipped with a flame ionization detector according to Lambert and Zitomer (1960). The volume of total gases produced during 24 h incubation was collected in 3 L Tedlar bags and measured through calibrated 60 mL plastic syringe (Dismadel SL, Madrid, Spain) according to Soliva and Hess (2007). MCP concentration was determined according to the modified optical density method Zhang et al. (2015). The NH_3_-N was analyzed by the indophenols method according to Weatherburn (1967). The pH values before or after feeding were measured immediately using pH electrode (Mettler, Toledo Ltd, England, and the UK). The nutrients disappearance rate was calculated according to Abbasi et al. (2018b).

The disappearance of DM and other indexes were calculated as (g/kg)

$$ {\text{Disappearance}} = \left[ {\left( {{\text{W}}_{ 3} {-}{\text{W}}_{ 4} } \right)/{\text{W}}_{ 3} } \right] \times 1000 $$

where, W_3_, % DM in the feed sample [(W_1_ − W) × DM%]; W_4_, residue DM weight [W_2_ − W] × 100; W, empty bag weight; W_1_, the weight of bag with feed sample before incubation; W_2_, the weight of bag with residue after incubation.

The metagenomic DNA in 4 mL of the fermenters fluid and 1 g undigested fermenters feed residue was extracted using a modified (CTAB) method as described by Kumar et al. (2014). The quality of the extracted DNA was detected using 1% agarose gel by electrophoresis and concentration of DNA was calculated at both absorbance ratio 260/280 and 230/260 by using spectrophotometer (Nano, Drop-2000 Thermo Technology, Inc, DE, USA). For further analysis DNA samples were stored at − 80 °C. The 16S rDNA genes copy number was determined through quantitative real-time PCR of cellulolytic specific-species*; F. succinogenes*, *R. albus*, *R. flavefaciens* and total bacteria. The PCR primer sets were used for amplification of total bacteria, and cellulolytic specific-species by (Koike and Kobayashi 2001; Schwiertz et al. 2010) were presented in Table 2.

**Table 2:** Primer sequence used for quantitative real-time PCR analyses

Target bacterial species	Forward primer sequence (5′-3′)	Reverse primer sequence (5′-3′)	Amplicon size (bp)
Total bacteria^a^	ACTCCTACGGGAGGCAGCAGT	ATTACCGCGGCTGCTGGC	174
*F. succinogenes* ^b^	GGTATGGGATGAGCTTGC	GCCTGCCCCTGAACTATC	446
*R. albus* ^b^	CCCTAAAAGCAGTCTTAGTTCG	CCTCCTTGCGGTTAGAACA	175
*R. flavefaciens* ^b^	TCTGGAAACGGATGGTA	CCTTTAAGACAGGAGTTTACAA	295

*F. succinogenes*, *Fibrobacter succinogenes*; *R. albus*, *Ruminococcus albus*; *R. flavefaciens*, *Ruminococcus flavefaciens*

^a^Schwiertz et al. (2010)

^b^Koike and Kobayashi (2001)

The copy number of each standard plasmid was calculated using as described by Yu et al. (2005), and the DNA standard calibration curve was made. The 16S rDNA copy numbers of cellulolytic species and total bacteria were calculated as by Zhao et al. (2013), and Liu et al. (2017). The real-time PCR was performed on ninety-six-well optical plates using CFX96TM Bio-Rad-IQ5 PCR System (Bio-Rad, Lab, Inc, Hercules, USA), was used for PCR assays with SYBR Green Dye Premix Ex Taq™II (Takara, Dalian, Liaoning, China). The PCR reaction (20 μL) contained 1 μL of forward and reverse primers, respectively, 7 μL of double standard sterile water, 1 μL of 30 ng (tenfold dilutions of 30 ng) of extracted bacterial genomic DNA, and 10 μL of SYBR Premix, Ex, Taq. The different copy numbers and cycle threshold (Ct) values were used to construct species-specific calibration curves. These curves were used for the calculation of DNA copy numbers (Zhao et al. 2013) all samples were examined in triplicate. The gene copy numbers were linearized by log_10_ before processing for data analysis.

### Data analyses

The data of 48 h incubation of all parameters of every incubation were averaged and each batch fermentation was considered as a replicate. The response variable was analyzed using IBM SPSS version 22.0 (SPSS Inc., Chicago, IL, USA) with treatment as a fixed factor and the incubation period was treated as a random factor. The probability value (*P*) and standard error of means (SEM) are presented in each Table. Significance was declared at (P < 0.05) and trends were discussed at (P < 0.10) differences among means were tested using Tukey’s multiple comparison tests.

## Results

### Effect of rumen-protected methionine supplements on the disappearance

The effects of supplements with low protein diet on disappearance indexes incubated 48-h in RUSITEC fermenters are presented in Table 3. Compared with the controls the disappearance of dry matter (DM), ether extract (EE) and acid detergent fiber (ADF) were unaffected by supplements of RPMet different levels (P > 0.05). However, NDF and GE degradation of supplement groups were non-significant to HP positive control, while significantly higher (P < 0.05) to the LP negative control group. Although, CP disappearance at RUSITEC fermenters was found significantly higher in supplement groups than HP and parallel with LP control groups.

**Table 3:** Effects of supplementation of rumen-protected methionine with low CP on disappearance using rumen simulation technique (n = 4)

Substrate disappearance	Treatments				SEM	P-value
	HP	LP	LPLMet	LPHMet		
Dry matter (g/kg DM)	693.28	696.78	692.03	700.44	11.67	1.00
Crude protein (g/kg DM)	773.94^b^	815.94^a^	824.01^a^	832.61^a^	7.75	0.01
Neutral detergent fiber (g/kg DM)	612.60^a^	534.69^b^	594.54^a^	611.40^a^	9.85	< 0.01
Acid detergent fiber (g/kg DM)	450.21	433.48	510.24	466.35	18.95	0.58
Ether extract (g/kg DM)	809.60	827.56	838.16	833.50	12.30	0.89
Gross energy (MJ/kg DM)	667.91^a^	602.08^b^	635.46^a^	663.74^a^	8.72	< 0.01

SEM, standard error of mean

^a,b^Superscripts values within the same row, are significantly different at (P < 0.05)

### Effect of the supplements on total and individual volatile fatty acids

The daily productions of total and individual VFA profiles were shown in Table 4. Daily production of acetate, propionate, butyrate (short-chain fatty acids), and the ratio of acetate to propionate were unaffected (P > 0.05) among LPHMet and HP group, but LPLMet was significantly (P < 0.05) lower than HP and similar (P > 0.05) to LP group. Total VFA production was linearly increased in LPHMet compared to LP but parallel to the HP group. Although, the molar proportion of LPLMet supplement was significantly higher than LP but lower than the HP group.

**Table 4:** Addition of RPMet with low CP their effects on daily production of total and individual volatile fatty acids using the RUSITEC (n = 4)

Items	Treatment				SEM	P-value
	HP	LP	LPLMet	LPHMet		
Total VFA (mM)	100.17^a^	87.42^c^	91.33^b^	100.47^a^	1.71	< 0.01
Acetate (mM)	50.74^a^	45.17^b^	47.17^b^	47.78^ab^	0.35	< 0.01
Propionate (mM)	27.60^a^	25.50^b^	25.83^b^	27.50^a^	0.32	< 0.01
Isobutyrate (mM)	0.83	0.81	0.82	0.83	0.36	0.99
Butyrate (mM)	13.67^a^	10.33^b^	11.07^b^	12.91^a^	0.45	< 0.01
Isovalerate (mM)	2.97	3.03	3.06	3.07	0.18	0.99
Valerate (mM)	4.18	3.65	3.89	4.09	0.02	0.79
Acetate: propionate ratio	1.84^ab^	1.75^b^	1.82^ab^	1.87^a^	0.02	0.04

VFA, volatile fatty acids; SEM, standard error of mean

^a,b,c^Superscripts values within the same row, are significantly different at (P < 0.05)

### Effect of the supplements on rumen pH, NH_3_-N, and microbial protein synthesis

Effects of RPMet supplements on rumen pH, MCP synthesis and NH_3_-N concentration from RUSITEC fermenters are presented in Table 5. The concentration of MCP of LPHMet was (P > 0.05) similar to HP and higher (P < 0.05) than the LP group. However, LPLMet was similar to LP and significantly lower than the HP group. The NH_3_-N production was unaffected (P > 0.05) among supplemented and LP, but it was significantly lower than the HP group. Compared with control groups supplementation of RPMet did not significantly alter rumen pH, the values before and after feeding on 0 to 24 h were found similar.

**Table 5:** Effects of rumen-protected methionine supplements on the synthesis of microbial crude protein, production of NH_3_-N and pH using a rumen simulation technique (n = 4)

Items	Treatments				SEM	P-value
	HP	LP	LPLMet	LPHMet		
pH before feeding*	6.65	6.67	6.66	6.68	0.01	0.83
pH after feeding (0–24 h)*	6.68	6.70	6.73	6.73	0.01	0.20
MCP (mg/mL)	3.18^a^	2.45^b^	2.62^ab^	3.10^a^	0.11	0.01
NH_3_-N (mg/100 mL)	18.43^a^	13.09^b^	13.39^b^	13.58^b^	0.80	0.02

MCP, microbial crude protein; NH_3_-N, ammonia nitrogen; SEM, standard error of mean

^a,b^Superscripts values within the same row, are significantly different at (P < 0.05)

* The pH values have published an article of the same first author

### Effect of the supplements on total and greenhouse gases production

Impact of RPMet supplements with low CP on total gas and greenhouse gases production are presented in Table 6. Production of methane gas was linearly decreased (P < 0.05) in supplemented groups during 24 h RUSITEC incubation than HP, but it was similar to (P > 0.05) LP group. While the volume of other gases; CO_2_ and H_2_ was unaffected compared to control groups. However, the total gas volume of HP control was significantly higher than the supplements and LP group.

**Table 6:** Effects of supplementation of RPMet with low CP on daily production and composition of gases using a RUSITEC technique (n = 4)

Items	Treatments				SEM	P-value
	HP	LP	LPLMet	LPHMet		
Total gas production (mL/day)	1435.75^a^	1390.75^b^	1381.25^b^	1377.00^b^	7.06	< 0.01
Individual gas production (mL/day)						
Hydrogen	4.88	3.65	3.80	4.10	0.25	0.31
Methane	302.47^a^	200.65^b^	205.29^b^	211.54^b^	11.83	< 0.01
Carbon dioxide	752.37	621.35	644.73	684.06	21.31	0.13

SEM, standard error of mean

^a,b^Superscripts values within the same row, are significantly different at (P < 0.05)

### Effect of the supplements on total and cellulolytic species 16S rDNA gene copy

The effect of supplements on the 16S rDNA gene copy numbers of total bacteria and cellulolytic species are presented in Table 7. The abundance numbers of 16S rDNA gene of cellulolytic species *R. albus* was similar among LPHMet and HP, but higher than the LP group. Whereas, LPLMet values were similar to (P > 0.05) LP and significantly lower (P < 0.05) than HP group at RUSITEC fermenters. Furthermore, compared to the control groups the16S rDNA gene copy numbers of total bacteria and other cellulolytic species *F*. *succinogene* and *R. flavefaciens* in supplements groups were found unaffected in liquid and a solid fraction at RUSITEC fermenters.

**Table 7:** Effects of experimental treatments on 16S rDNA gene copy numbers of three predominant ruminal and total bacteria from solid fractions using a RUSITEC (n = 4)

Items	Treatments				SEM	P-value
	HP	LP	LPLMet	LPHMet		
Solid fraction, log_10_ of 16S rDNA gene copy numbers per milliliter solid extracted liquid						
Total bacteria	11.79	10.43	10.93	11.90	0.35	0.45
*F. succinogenes*	8.31	6.99	7.82	8.52	0.27	0.20
*R. albus*	7.14^a^	5.96^b^	6.57^ab^	7.17^a^	0.16	< 0.01
*R. flavefaciens*	7.38	5.95	6.21	7.43	0.32	0.23
Liquid fraction, log10 of 16S rDNA gene copy numbers per milliliter fermenter liquid						
Total bacteria	9.83	8.44	9.00	10.04	0.33	0.30
*F. succinogenes*	6.23	5.74	5.99	6.73	0.17	0.18
*R. albus*	6.13^ab^	5.12b	5.40^ab^	6.21^a^	0.17	0.02
*R. flavefaciens*	5.31	4.40	4.71	5.71	0.28	0.40

SEM, standard error of mean

^a,b^Superscripts values within the same row, are significantly different at (P < 0.05)

## Discussion

In recent era numerous amino acids patterns for maintenance and production of dairy cows under investigation, the addition of simple AA in ruminants feed is not a sufficient choice to increase AA flow at the duodenum, because rumen atmosphere quickly degraded free AA (Volden et al. 1998). Rumen simulation is an advanced technique (RUSITEC) which simulate the condition of rumen in the laboratory circumstances under the strict control of saliva infusion, amount of feed, time of feeding, temperature, with allowing for measurement of rumen fermentation end products (Kajikawa et al. 2003). The present study is designed to measure which concentration of RPMet with low CP, reduce gases production and improve nutrients metabolism for further application in vivo study. To satisfy the maximum flow of AA in the small intestine to maintain dairy performance and also solve environmental problems related to high protein feeding in ruminant’s industry. The present study reported that the disappearance of CP was similar in supplement groups with LP and potentially higher than HP. However, the disappearance of NDF and GE were similar among LPHMet and HP group, while, LPLMet values were noted to be similar to the LP group. Based on a meta-analysis of Leonardi and Stevenson Armentano (2003) who reported that there were no differences in DMI either in low or high CP supplemented with RPMet. Also, Trinacty et al. (2009) noted that DMI (kg DM/day) of diets supplemented with RPMet was significantly higher than other diets. Noftsger et al. (2005) described that supplementation with RPMet to dairy cows improved the rumen fiber digestion and VFA production. Animals fed with high forage and maize-based ration balanced with RPMet either alone or in combination showed an increased digestibility of CP (Gajera et al. 2013). However, VFA production in the rumen did not depend only on the utilization rate but also depend on substrates, fiber degradation, bacterial populations and different sections of the reticulorumen (Bannink et al. 2006). The present study showed that the production of individual and total VFA from LPHMet diet was similar to HP group during 48 h incubation period. As previously discussed by Noftsger et al. (2005), the supplement of RPMet sources to the rumen had effects on rumen fermentation index (VFA, NH_3_-N). VanZijderveld et al. (2010) noted that VFA produced in the rumen by microbiological activity which degrades the cellulose and hemicellulose. However, Mulligan et al. (2002) reported that total and acetate VFA production associated with NDF degradability. In the current study, total gas and methane production were significantly decreased in RPMet supplementation groups. Raghavendra et al. (2007) and Yang et al. (2016) discussed that methane production depends on the fermentation of substrate, methanogens, ciliate protozoa, and hydrogenosomes, utilization of hydrogen, decline H_2_ production conversely decreasing CH_4_ production in the rumen. Furthermore, Kittelmann et al. (2014) who noticed the stronger associations between methane emissions and high population of H_2_ producing bacteria in sheep. Bannink et al. (2011) stated that the formation of VFA is followed by production of hydrogen gas and methanogenic archaea reduce CO_2_ and use H_2_ as a substrate for formation of propionate which decreases the overall CH_4_ production. Low CP supplemented with high methionine concentration promotes the synthesis of MCP which was similar to the HP group. The result of the present study corroborated with the findings of earlier studies. Reynolds and Kristensen (2008) concluded that overfeeding of protein in ruminant’s because problems, microbes or animal goes in the catabolism of protein or AA result in the conversion of the excess nitrogen, thus feeding low CP diet is appropriate to approach for MCP synthesis. In line with the study of VanSoest (1994) who discussed that MCP outflow improved in rumen when levels of structural fiber and NDF content of the ration passage increased because of microbes attached to fibrous particles. Wang et al. (2016) reported that the rate and extent of CP degradation and propionate production play a significant role in the MCP synthesis in the rumen. In the current study, ruminal NH_3_-N of LPLMet and LPHMet treatments was similar to the value of LP and ideal range for maximal MCP synthesis. Cole et al. (2005) and Todd et al. (2013) discussed that dietary intake and digestibility of CP influence NH_3_-N emissions by affecting N excretion. Furthermore, these results supported our findings according to Preston and Leng (1987), optimal ruminal NH_3_-N for efficient digestion was recorded 5.0 to 25.0 mg/dL. While, Weakley et al. (1983) reported that ruminal NH_3_-N from 9.34 to 11.23 mg/dL is acceptable for rumen bacterial metabolism and growth. In the current study, during simulation duration, the pH values observed unaffected before feeding or after feeding on 0 to 24 h in overall groups. As described in the previous studies by Calsamiglia et al. (2012), rumen utilizes different buffers and alkalizers and pH was recorded from 5.6 to 6.8. Furthermore, Russell and Wilson (1996) noted that ruminal pH ranges from 6.58 to 6.74 are suitable for the growth of cellulolytic bacteria in the rumen environment. Silva et al. (2016) reported that dietary CP levels did not affect ruminal pH in finishing beef cattle. Abbasi et al. (2018b) noted that different levels of RPMet with low CP could not significantly alter rumen pH before or after feeding. Furthermore, cellulose is the primary building component of plant matter microorganisms recycling them under anaerobic environment and extract energy (Vodovnik and Marinsek 2010). In the present study, the total bacterial population size of 16S rDNA gene copy numbers was found similar among all groups. The main cellulolytic spp. 16S rDNA copy numbers of; *R. flavefaciens*, and *F. succinogenes* were unaffected among all groups, but *R. album* population was found lower in LP than other groups. Previous studies noted that secondary metabolites of some ingredients enhance the growth of certain species of rumen microbes (Kim et al. 2014). Further, our findings agree with (Martin et al. 2013) who stated that rations which balanced for essential AA especially RPMet either alone or in combination increase significantly the production of fibrinolytic bacterial (*R. flavefaciens*, *F. succinogenes*, and *R. albus*) abundance. Furthermore, Yang et al. (2016) n that the main cellulolytic species *R. albus*, *R. flavefaciens* and *F. succinogenes* were not affected by dietary CP levels in lamb’s study. While, Russell and Wilson (1996) discussed that main cellulolytic species grow on cellulose, hemicellulose, pectin and growth depend on the production of is acids, fermentation products (acetate, butyrate, hydrogen and carbon dioxide). From the previous results, it was clear that the key role of RPMet supplementation to low protein diet on rumen fermentation characteristics is attributed to its indirect effect on the alteration of the ruminal environment that can significantly decrease rumen ammonia-N concentration which suggests that RPMet can promote ammonia utilization in the rumen. This result may be because RPMet supplementation can enhance rumen microbial growth and the microbes can reutilize degraded ammonia-N to synthesize microbial proteins (Or-Rashid et al. 2001). Also, dietary RPMet supplementation leads to high RUP:RDP ratio which in turn improves the healthy condition of the rumen. The indirect effect of RPMet could enhance the growth and multiplication of cellulytic microbes for efficient DM digestion, production of volatile fatty acids and stimulation of microbial protein synthesis. Improvement of these parameters had a positive impact on productive and reproductive performance, feed cost as well as environmental pollution.

Menchu (2019) concluded that *N*-acetyl-l-methionine and *N*-acetyl-l-lysine have potential to provide rumen protection; and determining the optimum dosages will help to increase the benefit of these products. There also appears to be potential to increase rumen fermentation products, which is important for maintaining high milk production in dairy cows.

The dietary supplementation of RPMet at a level of (0.11 g/kg) the most of the studied parameters was unaffected at RUSITEC. At a level of (0.81 g/kg) inclusion, the MCP, short chain fatty acids, CP, NDF, GE, and *R. albus* population were improved. Furthermore, the production volume of total and methane gas was lower in supplement groups. In future, it should be emphasized the promising RPMet level to be tested in vivo study to evaluate effects on the flow of AA as MP at the small intestine with productive and reproduction performance, co-occurrence patterns of the microbiome in the rumen, which shines new light on fermentation, their interaction, and gases emission.

## Data Availability

Not applicable.

## Abbreviations

AA: amino acids
ADF: acid detergent fiber
CP: crude protein
GE: gross energy
RPMet: rumen-protected methionine
HP: high protein diet (163.39 g/kg CP) without RPMet
LP: low protein diet (146.33 g/kg CP) without RPMet
LPHMet: low protein diet, supplement with high RPMet (RPMet: 0.81 g/kg)
LPLMet: low protein diet, supplement with low RPMet (RPMet: 0.11 g/kg)
MCP: microbial crude protein
MP: metabolizable protein
NDF: neutral detergent fiber
NH3-N: ammonia-N
NRC: National Research Council
RDP: rumen degradable protein
RUP: rumen undegradable protein
RUSITEC: rumen simulation technique
VFA: volatile fatty acid
